# Cancer-derived extracellular vesicles: friend and foe of tumour immunosurveillance

**DOI:** 10.1098/rstb.2016.0481

**Published:** 2017-11-20

**Authors:** Bastian Dörsam, Kathrin S. Reiners, Elke Pogge von Strandmann

**Affiliations:** 1 Experimental Tumor Research, Center for Tumor Biology and Immunology, Clinic for Hematology, Oncology and Immunology, Philipps University, Hans-Meerwein-Street 3, 35043 Marburg, Germany; 2 Institute of Clinical Chemistry and Clinical Pharmacology, Biomedical Center, University Hospital, University of Bonn, Sigmund-Freud-Street 25, 53127 Bonn, Germany

**Keywords:** tumour immunology, innate immunity, natural killer cells, extracellular vesicles

## Abstract

Extracellular vesicles (EVs) are important players of intercellular signalling mechanisms, including communication with and among immune cells. EVs can affect the surrounding tissue as well as peripheral cells. Recently, EVs have been identified to be involved in the aetiology of several diseases, including cancer. Tumour cell-released EVs or exosomes have been shown to promote a tumour-supporting environment in non-malignant tissue and, thus, benefit metastasis. The underlying mechanisms are numerous: loss of antigen expression, direct suppression of immune effector cells, exchange of nucleic acids, alteration of the recipient cells' transcription and direct suppression of immune cells. Consequently, tumour cells can subvert the host's immune detection as well as suppress the immune system. On the contrary, recent studies reported the existence of EVs able to activate immune cells, thus promoting the tumour-directed immune response. In this article, the immunosuppressive capabilities of EVs, on the one hand, and their potential use in immunoactivation and therapeutic potential, on the other hand, are discussed.

This article is part of the discussion meeting issue ‘Extracellular vesicles and the tumour microenvironment’.

## 1. Introduction

Extracellular vesicles (EVs) were initially described a few decades ago; all cells release certain membrane vesicles with a great variety of important functions. In 1984, vesicle release was described as a novel mechanism of transferrin receptor secretion in sheep reticulocytes [1]. This release is linked to the formation of intracellular exosomes, originating from an endosomal multi-vesicular body (MVB), which fuse with the cells plasma membrane [2].

Originally, budding of vesicles from the plasma membrane was suggested to be part of the lysosomal degradation pathway, responsible for the excretion of cell debris [3] and emergency membrane repair [4]. Subsequent studies drew attention to the role of B lymphocyte-secreted EVs in regulation of the immune response [5] and, about a decade later, intercellular exchange of mRNAs and miRNAs via EVs was confirmed by Valadi *et al*. [6].

Cells produce and release different types of EVs, which can be distinguished according to their size: apoptotic bodies (1000–5000 nm) characterize the largest fraction, microvesicles (200–1000 nm) comprise the intermediate fraction and exosomes (30–150 nm) are the smallest fraction [7]. Exosomes are ubiquitously released by all cells, including malignant cells, and are present in the body fluids [8]. In contrast with other EVs, the biogenesis of exosomes starts with an invagination of the plasma membrane. During maturation, the initial endosome experiences several inward invaginations forming numerous intraluminal vesicles and thus incorporating components of the cytosol. The endosome becomes a so-called MVB comprising multiple vesicles which contain different proteins and nucleic acids [9]. MVBs can subsequently fuse with the plasma membrane releasing the contained exosomes into the extracellular space. Apart from that, MVBs can enter the lysosomal degradation pathway. The fate of the MVBs is dependent on the amount of ceramides contained in the membrane-associated lipids [7]. The exosomes released in this manner carry a characteristic and cell type-specific composition of nucleic acids, proteins, enzymes, lipids, cytokines and other soluble factors inherited from the parental cell [10,11]. The endosomal sorting complex required for transport (ESCRT) is responsible for packing and trafficking of exosomes or subtypes of exosomes [12]. During this process, exosomes are loaded with components of the ESCRT and associated molecules [13,14], which are common markers used to identify exosomes of endocytic origin [7,11]. These molecules include parent cell-characteristic annexins, flotillin, GTPases, lipids and cholesterol [15–17], as well as tetraspanins (CD9, CD63, CD81, CD82) [18,19] and proteins of the accessory ESCRT pathway (e.g. ALIX and TSG101) [13]. Although the content of the exosomes does not completely resemble the profile of the parental cell, the partial similarity inspired the idea of using exosomes as biomarkers for tumours. Differences in the profile of parent cells and exosomes indicate the participation of still unknown processes [20,21]. Besides the ESCRT, other sorting mechanisms dependent on raft-based microdomains have been proposed to be involved in the genesis of exosomes [22,23]. Apart from the exosome fraction, certain microvesicles, the so-called ectosomes, can be formed by membrane blebbing [9]. These EVs are also suggested to play a role in intercellular communication. Yet the differentiation between exosomes and microvesicles is not completely understood [9]. This challenges the use of vesicle size as reliable indicator for the definition of EVs and both fractions need to be analysed to identify suitable EV-associated biomarkers [20].

Composition, biogenesis and secretion of EVs/exosomes are adaptive processes influenced by extrinsic stimuli including cellular stress. Cells are able to respond to intracellular stress situations by secretion of vesicles influencing their environment [24]. Moreover, they play an important role in the host's immune response. Among others, dendritic cell-derived exosomes (Dex) are involved in the immune system's response to tumours and promote the proliferation and cytolytic activity of natural killer (NK) cells [25]. Malignant cells are frequently challenged with stress situations such as hypoxia, starvation or chemotherapeutic drugs in their microenvironment which they need to overcome to facilitate progression of the tumour [24]. It is well known that tumours shape their microenvironment by EV/exosome-mediated communication with the surrounding stromal tissue, thus promoting proliferation, angiogenesis, suppression of the host's immune defence and initiation of pre-metastatic niches [26]. Further, the release of tumour-derived EVs/exosomes (T-EVs) is frequently increased in tumour patients [27] and especially elevated after chemotherapy or photodynamic treatment [28]. Interestingly, the tumour suppressor p53, which is tightly connected to the aetiology of cancer, is involved in the regulation of vesicle release [29]. Protein microarray analysis of peripheral blood mononuclear cells (PBMCs) revealed an immunosuppressive effect of T-EVs at high concentrations, whereas PBMCs showed an activated phenotype at low concentrations [30]. In line with the latter, T-EVs can also carry so-called tumour-associated antigens (TAAs), costimulatory molecules and major histocompatibility complexes (MHC) components mediating a stimulatory effect on immune cells [31,32]. These findings suggest a switch in the virtue of EVs from immunoactivation towards immunosuppression during tumour progression. To date, the underlying molecular basis for this functional alteration remains largely elusive.

## 2. Immunosuppression by cancer-derived extracellular vesicles

T-EV-mediated communication is likely to benefit the tumour's progression and survival. During their progression, tumours develop several T-EV-based approaches to interfere with the host's immune system counteracting anti-tumour activities. This requires some sort of interaction between T-EVs and immune cells such as binding or internalization of the vesicles [33]. Ligands or antigens present in or on T-EVs can directly interact with receptors on lymphocytes or bind to cellular MHC receptors, respectively. Receptor-mediated uptake allows T-EVs to fuse with the cell's plasma membrane and release their contents into the cytoplasm. In addition, phagocytic cells (e.g. macrophages and dendritic cells; DCs) can easily internalize T-EVs. T-EVs interacting with surface molecules on T-cells transfer signals and by this alter their function [34]. To bypass the host's immune response, tumours subvert the recognition by cytotoxic T-lymphocytes (CTLs), impair the antigen presentation by antigen-presenting cells or interfere with the host's immune response. Moreover, immune cells can be tricked to support the tumour. In these strategies, the appropriate surface proteins, intravesicular cytokines or nucleic acids, with which EVs are equipped, play a crucial role [35].

T-EVs containing so-called death ligands, e.g. Fas ligands or tumour necrosis factor-α (TNF-α), hold the potential to directly induce cell death in immune cells through activation of the death receptor family members TNF receptor 1 (TNF-R1) and Fas receptor (FasR), respectively. Activation of these receptors is tightly linked to the induction of necrosis and caspase-dependent cell death [36–40].

One strategy of immune evasion is direct EV-mediated immune suppression. The primary target of this strategy are the CTLs. The potential of T-EVs to inhibit the growth of CD8^+^ CTLs is reported for several cancer types [38,41]. Transforming growth factor-β (TGF-β) is one of the most prominent immunosuppressive cytokines found on the surface of EVs. Suppression of NK cell function and T-cell proliferation by vesicular TGF-β on T-EVs was observed in patients suffering from acute myeloid leukaemia [27] and breast cancer [42]. Peinado *et al*. [43] demonstrated the potential of T-EVs derived from highly metastatic melanomas to reprogramme bone marrow cells to form a melanoma-friendly environment. Thus, T-EVs are able to interfere with the development and differentiation of haematopoietic cells as well as with the functions of mature cells [44,45]. Additional to direct suppression and cell death induction, T-EVs can induce the differentiation of regulatory T cells (Tregs) and myeloid-derived suppressor cells [38,46].

Host cells express MHC-I molecules, protecting them from the attack of CTLs, whereas tumour cells expressing MHC-I/TAA complexes are destroyed by CTLs. Downregulation of the MHC-I/TAA complexes allow the tumour to escape detection by the adaptive immune system [47]. However, cells lacking the MHC-I complex are approached and eliminated by NK cells [48]. To avoid the attack of NK cells, tumours are able to release EVs influencing the cytotoxic activity of NK cells [49], which is regulated by an equilibrium of activating and inhibitory receptors. The ligands of the NK cell-activating receptor NK group 2, member D (NKG2D) MHC class I chain-related proteins A and B (MIC-A and MIC-B) and UL-16-binding protein [50] are present on the surface of EVs [51]. EVs bearing NKG2D ligands act as bait for NK cells by distracting the immune cells from the tumour [51]. Additionally, these EVs elicit a downregulation of NKG2D receptors on NK cells [52,53]. Owing to the high proliferation rate of many tumour cells, the tumour is likely to outgrow the blood supply, resulting in large parts of the tumour tissue being supplied with low oxygen concentrations. In order to survive in the hypoxic microenvironment, tumour cells are known to adapt their metabolism [54]. A study published by Berchem *et al*. proved that T-EVs emerging from hypoxic conditions had a stronger inhibitory impact on NK cells compared to T-EVs originating from normoxic conditions. The increased immunosuppressive potential was attributable to the transfer of miR-23a and TGF-β to NK cells [55]. In addition, increased levels of miR-4498 were observed in hypoxic T-EVs derived from melanoma cells [35]. CD83, which is a key in the communication between cells of the innate and adaptive immune response, is regulated by miR-4498 [56].

*In vitro* studies indicate the intercellular exchange of nucleic acids via EVs [6,57]. Ding *et al*. demonstrated an increase in cancer-related miRNAs as well as inhibition of a wealth of mRNAs in DCs exposed to pancreatic cancer-derived T-EVs. Interestingly, the authors revealed an inhibition of the MHC II transcription factor regulatory factor X-associated protein (RFXAP) by miR-212-3p received from T-EVs. This was further confirmed by clinical data negatively correlating miR-212-3p and RFXAP in pancreatic cancer tissue [58]. The presence of inhibitory miRNAs or mRNAs promoting the aetiology of cancer and negatively influencing the host's immune response was also suggested for T-EVs derived from other cancer species [59,60]. A recent study stated that T-EV-recipient cells experience a regulation of genes responsible for the immune response [61]. In detail, gene profiles of several human T-cell subsets exposed to T-EVs *in vitro* were analysed. Tregs were most sensitive to EV-mediated effects and experienced downregulation of genes involved in the adenosine pathway, which induces a high expression of CD39 and enhanced adenosine production [61]. Extracellular ectonucleotidases such as CD39 contribute to high levels of the purine nucleoside adenosine [62], which is a powerful immune regulator attenuating local immune responses [63]. Besides, T-EVs caused an upregulation of inhibitory genes in CD4^+^ T cells that are responsible for loss of function via downregulation of CD69 expression. T-EVs carrying the ectonucleosidases CD73 and CD39 on their surface can, moreover, produce extracellular adenosine, directly interfering with T cells [64]. Concomitant with induction of necrosis, TNF-containing T-EVs from melanoma cells induce the production of intercellular reactive oxygen species in T-cells, which impairs the T-cell receptor signalling pathway and hence leads to a decrease in T cells [65].

## 3. Extracellular vesicle-mediated immunoactivation

Apart from the critical immunosuppressive potential of T-EVs, vesicles bearing immune-activating effects have been described recently. This mirrors the diverse and differentiated functions of EVs. Latest research has focused on the immunostimulatory properties of dendritic cell-derived exosomes (Dex) and their potential value for immunotherapy [66,67]. Dex maintain the central functions of DCs: presentation of TAAs and activation of TAA-specific immune responses. Their membrane harbours a variety of molecules responsible for antigen presentation (MHC class I, class II, CD1), adhesion (intercellular adhesion molecules), costimulatory signals (CD86, CD40) and docking (integrins) [68,69].

Viaud *et al*. demonstrated that Dex promote an interleukin-15 Rα- (IL-15Rα) and NKG2D-dependent proliferation and activation of NK cells in a murine *in vivo* model, resulting in an anti-metastatic effect. Furthermore, human Dex are equipped with NK cell-activating NKG2D ligands. A Dex-based vaccine was able to restore NKG2D-dependent functions of NK cells in half of the tested melanoma patients [25].

The melanoma-associated tumour antigens (MAGEs) are usually not present on host cells but are commonly expressed by different tumour species [70]. An early phase I clinical study addressing the therapeutic use of MAGE antigen-loaded Dex in 15 MAGE3^+^ advanced melanoma patients reported a response in one patient, one minor response and two stabilizations of disease. Although almost two-thirds of patients showed NK cell effector functions, no MAGE-specific T-cell responses were observed in the peripheral blood [71]. In a second phase I clinical trial performed by another group, one-third of advanced MAGE^+^ non-small cell lung cancer (NSCLC) patients developed MAGE3.A1-specific systemic immune responses in line with upregulation of NK cell activity [72]. TAA-loaded Dex have proven their feasibility of large-scale production and outstanding safety profile in these studies [71,72]. In contrast with Dex from immature DCs, new approaches using EVs derived from TLR4 L- or interferon (IFN)-γ-maturated DCs showed improved Dex-induced T-cell stimulation [73–76]. A recent phase II clinical trial applying IFN-γ-Dex loaded with MHC class I- and class II-restricted cancer antigens as immunotherapy of NSCLC patients after chemotherapy showed that the expression of MHC class II on Dex correlated with the expression of the NK cell activating NKp30 ligand BCL2-associated athanogene 6 (BAG6) [77]. The chaperone BAG6 plays a role in a multitude of cellular processes and was identified as ligand of the activating NK cell receptor NKp30 [78,79]. The expression patterns of both BAG6 and MHC-II are tightly connected and controlled by the IFN-γ-inducible class II transactivator (CIITA) [80]. BAG6 is necessary for the accumulation of HSP70 [81,82], which bears the potential to activate the immune response. HSP70 induces the maturation of DCs and promotes the phagocytosis of tumour cells as well as cross-presentation of chaperoned peptides. NK cells are required for the interaction of DCs and HSP70 to induce a CTL response and anti-metastatic effect *in vivo* [83]. Moreover, HSP70/BAG4 surface-positive T-EVs specifically facilitate migration and HSP70 reactivity of NK cells. HSP70-specific antibodies can inhibit the T-EV-induced cytolytic activity of NK cells [84]. It should be noted that a soluble form of BAG6 (sBAG6) in the plasma of chronic lymphocytic leukaemia patients critically impaired the function of NK cells [85], whereas vesicular BAG6 is a powerful activator of NK cells [86]. According to Besse *et al*. [77], a possible explanation for the opposing virtue of EV-BAG6 and sBAG might be the interplay of BAG6 and HSP70 on exosomes to activate NK cells via co-engagement of NKp30 and a second regulatory NK cell receptor, CD94, also known as NKG2. An upregulation of CD94 receptors on NK cells is described to correlate with enhanced cytolytic activity after stimulation with HSP70 or HSP70 and IL-2 [87,88]. Alternatively, the oligomerization of EV-BAG6 was discussed as possible explanation for the contrasting activities of EV-BAG6 and sBAG6 [79]. The cytosolic immune-sensing receptor retinoic acid-inducible gene I (RIG-I) is ubiquitously expressed in nucleated cells, including malignant cells [89], and activated by viral 5′-triphosphorylated RNA [90,91]. Dassler-Plenker *et al*. discovered a novel mechanism of RIG-I-mediated release of EVs with anti-tumour activity from tumour cells. The EVs showed increased expression of BAG6 on their membrane, thus activating NKp30-mediated cytotoxic activity of NK cells [92].

Besides BAG6, B7-H6, which is a member of the B7 family of immunoreceptors, is a well-known cell surface ligand for the NK cell-activating receptor NKp30 [93,94]. In contrast to BAG6, the expression of B7-H6 is restricted to tumour cells [93,95]. B7 family members are induced on myeloid cells upon inflammatory stimuli [96,97], but the underlying mechanism remains unresolved. Matta *et al*. discovered B7-H6 in the vesicle fraction after ultracentrifugation, indicating that B7-H6 could be included in EVs present in patients' serum or be present as soluble variant. *In vitro* experiments revealed that the isolated B7-H6 originated from activated monocytes and neutrophils and possessed the potential to modulate the activity of NK cells [98].

## 4. Conclusion and future challenges

**Figure 1.**
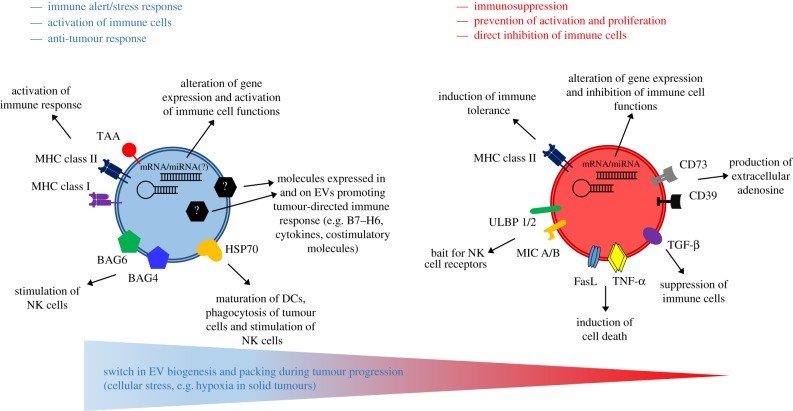
Scheme of EVs carrying immunoactivating and immunosuppressing molecules and their impact on the immune response. Future research identifying yet unknown molecules expressed in and on the surface of EVs might clarify the mechanism underlying the switch from immune altering EVs to immunosuppressing T-EVs during tumour progression. Moreover, new biomarkers for tumours and therapeutic approaches enhancing the host's tumour-directed immune response could be envisioned.

Tumour cell-derived EVs may either trigger or, on the contrary, suppress anti-tumour immune responses and their biological role is controversial (figure 1). Some molecules expressed on immune-activating or suppressive EVs are indicated; however, the plasticity of T-EVs or differences in EV subtypes remain to be investigated. This analysis will enable us to identify the cargo (including nucleic acids, lipids and proteins) which is responsible for the functional activity of EVs or of a given EV subtype. There is emerging evidence that DNA damage or stimuli of the microenvironment such as hypoxia or receptor activation impact on EV biosynthesis, cargo loading or their release. A better molecular understanding of the downstream pathways directing EV composition and secretion is mandatory for the rational therapeutic application of EVs to combat cancer.

## Data accessibility

This article has no additional data.
